# Editorial: Biogenic volatiles in natural and urban forest

**DOI:** 10.3389/fpls.2023.1233612

**Published:** 2023-07-19

**Authors:** Cecilia Brunetti, Barbara Baesso Moura, Violeta Velikova

**Affiliations:** ^1^ Institute for Sustainable Plant Protection (IPSP), National Research Council, Sesto Fiorentino, Italy; ^2^ Institute of Research on Terrestrial Ecosystems (IRET), National Research Council, Sesto Fiorentino, Italy; ^3^ National Biodiversity Future Center (NBFC), Palermo, Italy; ^4^ Institute of Plant Physiology and Genetics, Bulgarian Academy of Sciences, Sofia, Bulgaria

**Keywords:** BVOCs, abiotic stresses, biotic stresses, climatic factors, secondary metabolites

Biogenic volatile organic compounds (BVOCs) are secondary metabolites which play an important role in the adaptive capacity of trees in response to climate change. BVOCs released into the atmosphere, including isoprene, monoterpenes, sesquiterpenes, and their oxygenated derivatives, are shaping the oxidative capacity of the atmosphere, particularly within forested regions (Faiola et al., 2012), thus contributing to the regulation of the global climate. Substantial research has been devoted to BVOC emissions and their involvement in atmospheric chemistry and plant physiological processes (Peñuelas and Llusià, 2003; Bao et al., 2022). The main functions of BVOCs are summarized in **Figure 1**.

**Figure 1:**
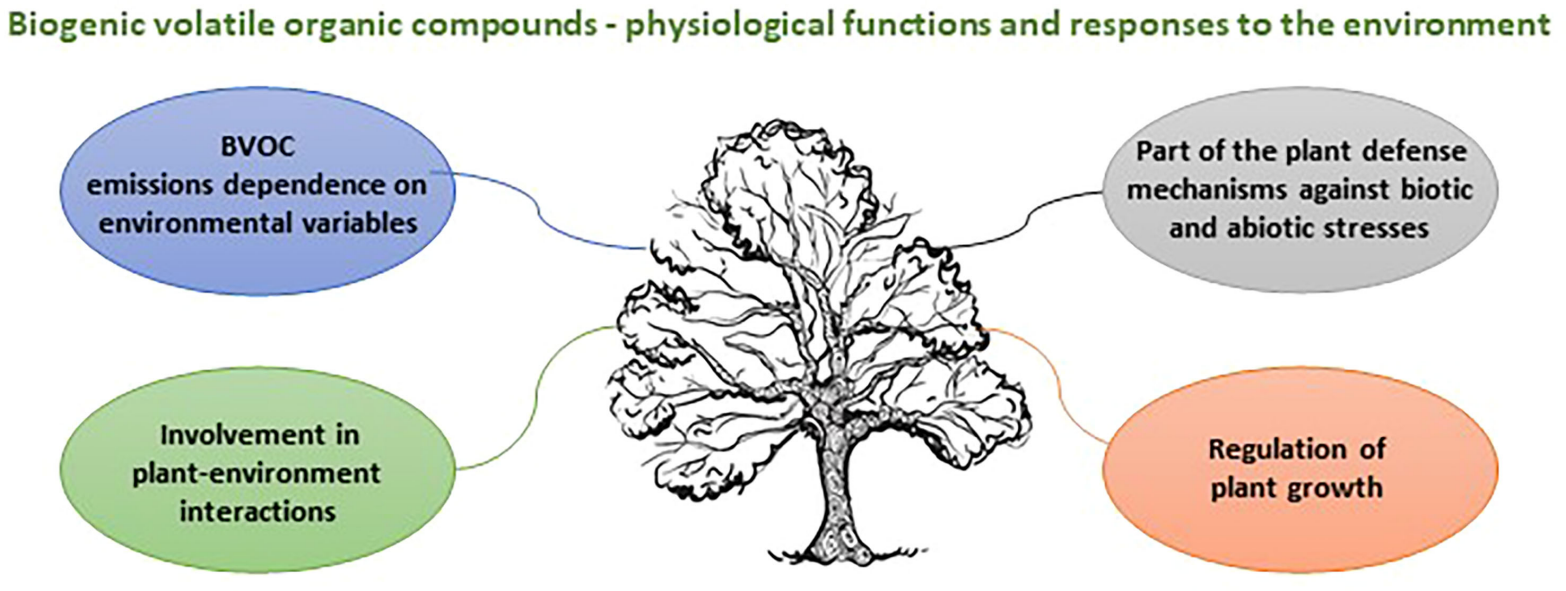
Main functions of BVOCs in plant-environment interactions.

BVOC emissions are involved in defense mechanisms against biotic stresses, such as herbivores and parasites, acting both as allelochemicals and as as signals to neighboring plants (Kegge and Pierik, 2010). They play a major role in plant-to-plant communication, functioning as growth modulators and as signals about herbivory or other stress stimuli, enabling neighboring plants to activate their own defense mechanisms (Ninkovic et al., 2021). BVOCs are also involved in responses to abiotic stresses such as drought, high temperatures and air pollution, enhancing plant tolerance and resilience to various environmental stresses (Loreto and Schnitzler, 2010).

The Research Topic “*Biogenic Volatiles in Natural and Urban Forest*” comprises one review article and four research papers.

The blend of BVOCs is species-specific and constitutes a metabolic fingerprint than can be deciphered through volatilomic screening (Majchrzak et al., 2020). The amount of emissions is greatly influenced by environmental conditions and phenological stages. The review article by Mu et al. conducted an in-depth analysis of the spatial and temporal fluctuations of these emissions. It is emphasized on the regional variability and species specificity of the biogenic emissions.

The emission of BVOCs from tropical plants is also the topic of the paper of Moura et al. who investigated the effect of ozone (O_3_) on three native species from the Atlantic Rain Forest: *Croton floribundus*, *Astronium graveolens* and *Piptadenia gonoacantha*. By combining measurements of BVOC emissions and histochemical techniques, the authors aimed to reveal the strategies of these species to counteract oxidative stress. Moreover, the authors discussed the interaction of BVOCs emissions with the atmospheric chemistry, particularly within the formation of tropospheric O_3_, which in turn could stimulate further BVOCs emissions.

Since the emission of BVOCs also varies on the basis of the microclimatic conditions, Tullus et al. investigated the effects of competitive status, within-crown light environment, and climate on the secondary chemistry of *Betula pendula*. The investigation conducted by the researchers revealed notable fluctuations in the composition and profile of secondary metabolites, primarily related to phenolic defense compounds and growth regulators, in response to climatic factors. These variations align with the trees’ adaptive defense mechanisms against herbivory, exposure to irradiance, and competitive status in terms of resource availability. Furthermore, the metabolic profile exhibited by the fine roots not only corresponds to defense requirements, but also signifies distinct below-ground competition strategies in warmer and colder climates.

Finally, two studies investigated the constitutive emissions of plants. Effah et al. investigated BVOCs emission pattern of the shrub *Dracophyllum subulatum*, a species native in the Central Plateau of the North Island, New Zealand, emphasizing on the relationship between emissions induced by biotic and abiotic factors. Fitzky et al. aimed to provide insights into the species-specific stress tolerance potential of seedlings of four broadleaf tree species: *Quercus robur*, *Fagus sylvatica*, *Betula pendula*, and *Carpinus betulus*. This study shows that the emissions of specific BVOCs are highly interrelated and can be considered the first step in linking the metabolism and function of co-occurring BVOCs emissions.

In conclusion, the papers published combine a series of new valuable information for readers in the field of BVOC emissions - atmospheric chemistry interactions, as well as the fluctuations in secondary metabolites and their adaptive defense mechanisms in response to climatic factors. Additional studies on emission patterns under stress conditions will be of utmost significance to better understand plant-environmental interactions in the face of intensifying climate change. In addition, owing to high air pollution concentrations, prospective changes in BVOC emission blends in urban areas merit special consideration.

## Author contributions

All authors listed have made a substantial, direct, and intellectual contribution to the work and approved it for publication.

## References

[B1] Bao X. Zhou W. Xu L. Zheng Z. (2022). A meta-analysis on plant volatile organic compound emissions of different plant species and responses to environmental stress. Environ. pollut. 318, 120886. doi: 10.1016/j.envpol.2022.120886 36549454

[B2] Faiola C. L. Erickson M. H. Fricaud V. L. Jobson B. T. Vanreken T. M. (2012). Quantification of biogenic volatile organic compounds with a flame ionization detector using the effective carbon number concept. Atmos. Measurement Techniques 5 (8), 1911–1923. doi: 10.5194/amt-5-1911-2012

[B3] Kegge W. Pierik R. (2010). Biogenic volatile organic compounds and plant competition. Trends Plant Sci. 15 (3), 126–132. doi: 10.1016/j.tplants.2009.11.007 20036599

[B4] Loreto F. Schnitzler J. P. (2010). Abiotic stresses and induced BVOCs. Trends Plant Sci. 15 (3), 154–166. doi: 10.1016/j.tplants.2009.12.006 20133178

[B5] Majchrzak T. Wojnowski W. Rutkowska M. Wasik A. (2020). Real-time volatilomics: a novel approach for analyzing biological samples. Trends Plant Sci. 25 (3), 302–312. doi: 10.1016/j.tplants.2019.12.005 31948793

[B6] Ninkovic V. Markovic D. Rensing M. (2021). Plant volatiles as cues and signals in plant communication. Plant Cell Environ. 44 (4), 1030–1043. doi: 10.1111/pce.13910 33047347 PMC8048923

[B7] Peñuelas J. Llusià J. (2003). BVOCs: plant defense against climate warming? Trends Plant Sci. 8 (3), 105–109. doi: 10.1016/S1360-1385(03)00008-6 12663219

